# Hippocampal phase precession is preserved under ketamine, but the range of precession across a theta cycle is reduced

**DOI:** 10.1177/02698811231187339

**Published:** 2023-07-29

**Authors:** Lucinda J Speers, Daena J Sissons, Lana Cleland, David K Bilkey

**Affiliations:** 1 Psychology Department, Otago University Dunedin, New Zealand; 2 Psychology Department, University of Canterbury, Christchurch, New Zealand; 3 Department Psychological Medicine, Otago University, Christchurch, New Zealand; 4 Department Population Health, Otago University, Christchurch, New Zealand

**Keywords:** Phase precession, ketamine, hippocampus, theta oscillations, schizophrenia

## Abstract

**Background:**

Hippocampal phase precession, which depends on the precise spike timing of place cells relative to local theta oscillations, has been proposed to underlie sequential memory. N-methyl-D-asparate (NMDA) receptor antagonists such as ketamine disrupt memory and also reproduce several schizophrenia-like symptoms, including spatial memory impairments and disorganized cognition. It is possible that these impairments result from disruptions to phase precession.

**Aims/methods:**

We used an ABA design to test whether an acute, subanesthetic dose (7.5 mg/kg) of ketamine disrupted phase precession in CA1 of male rats as they navigated around a rectangular track for a food reward.

**Results/outcomes:**

Ketamine did not affect the ability of CA1 place cells to precess despite changes to place cell firing rates, local field potential properties and locomotor speed. However, ketamine reduced the range of phase precession that occurred across a theta cycle.

**Conclusion:**

Phase precession is largely robust to acute NMDA receptor antagonism by ketamine, but the reduced range of precession could have important implications for learning and memory.

## Introduction

Schizophrenia is a chronic neurodevelopmental disorder with complex pathophysiology and diverse symptoms that can include hallucinations and delusions (positive symptoms), avolition and flattened affect (negative symptoms), and cognitive dysfunction such as deficits in spatial memory and planning processes. Some of these positive, negative and cognitive symptoms can be transiently induced in healthy individuals following acute exposure to the NMDA receptor antagonists phencyclidine and ketamine (Beck et al., 2020; Javitt and Zukin, 1991; Krystal et al., 1994; Moghaddam and Javitt, 2012; Umbricht et al., 2000). These findings have provided support for the glutamate hypothesis of schizophrenia, which proposes that dysfunction in NMDA-mediated neurotransmission creates an imbalance of glutamate and γ-aminobutyric acid (GABA) signaling in several key regions known to be involved in schizophrenia pathophysiology, including the hippocampus (Frohlich and Van Horn, 2014; Moghaddam and Javitt, 2012; Nakazawa et al., 2017; Uno and Coyle, 2019). Ketamine may mimic this imbalance via selective antagonism of inhibitory interneurons that results in cortical disinhibition or by direct inhibition of pyramidal neurons (Miller et al., 2016).

The hippocampus plays a critical role in both spatial and episodic memory (O’keefe and Nadel, 1978; Tulving and Markowitsch, 1998), as well as sequential planning (Buzsáki and Tingley, 2018; Foster and Knierim, 2012), and these processes are all known to be disturbed in individuals with schizophrenia (Leavitt and Goldberg, 2009; Piskulic et al., 2007; Semkovska et al., 2004). Spatial memory deficits have also been observed in humans with either a history of ketamine use (Morgan et al., 2014) or following acute exposure (Morgan et al., 2004). In rodent models, subanesthetic doses of ketamine have been shown to produce deficits in spatial, working and episodic-like memory following both acute (Boultadakis and Pitsikas, 2010; de Souza et al., 2019; Duan et al., 2013; Pitsikas et al., 2008) and chronic exposure protocols (Venâncio et al., 2011). At the physiological level, acute ketamine exposure induces changes in both theta, gamma and high-frequency (High Frequency oscillation (HFO) ~100–180 Hz) oscillations in several brain regions including the hippocampus and prefrontal cortex (Caixeta et al., 2013; Kittelberger et al., 2012; Moran et al., 2015; Nottage et al., 2023; Olszewski et al., 2013), as well as inducing synaptic depression in CA1 (Duan et al., 2013). Chronic ketamine administration has also been shown to reduce the density of PV interneurons in the hippocampus (Kittelberger et al., 2012; Koh et al., 2016), mirroring reports of PV interneuron disruptions in both individuals diagnosed with schizophrenia (Kaar et al., 2019; Yanagi et al., 2014) and animal models of the disorder (Lewis et al., 2012; Lodge et al., 2009).

Spatial memory and planning are thought to require the sequential spiking of hippocampal place cells (Dragoi and Buzsáki, 2006; Foster and Knierim, 2012; Jaramillo and Kempter, 2017) at temporal scales suitable for the induction of synaptic plasticity (Bi and Poo, 1998). Current evidence suggests phase precession of cell spiking may be one of the mechanisms that underlies this process (Dragoi and Buzsáki, 2006; Jaramillo and Kempter, 2017). Phase precession is a robust phenomenon in which the spiking phase of pyramidal cells systematically advances relative to the background local field potentials (LFP) theta oscillation as an animal runs across a place field (O’Keefe and Recce, 1993; Skaggs et al., 1996). Neurons thus translate location information into a phase code, which allows for the generation of “theta sequences” across a population of cells with overlapping place fields, whereby the sequential order of place field position is compressed within a single theta cycle (~120 ms) (Foster and Wilson, 2007). Importantly, organized theta sequences will only occur if the starting phase of precession, occurring as an animal enters a new field, or the rate of precession (as measured by the precession slope) is relatively consistent across a cell assembly (Feng et al., 2015; Schmidt et al., 2009).

**Figure 1.**
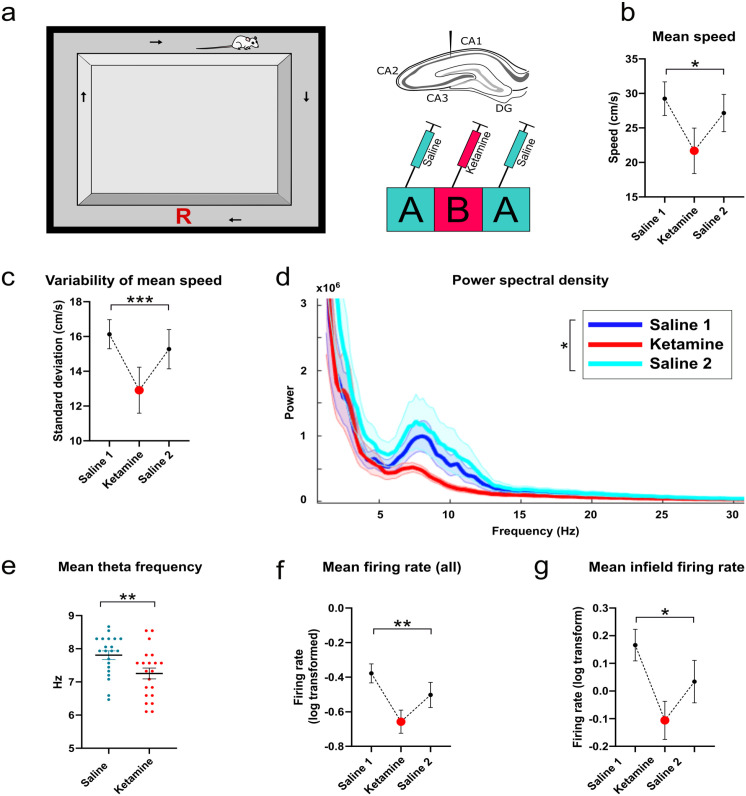
Schematic of experimental design, and data describing locomotor speed, LFP properties and firing rates: (a) Rats were trained to run clockwise around a rectangular running track, with a food reward delivered manually in the region marked with a red “R”. To the right is a diagram of approximate electrode placement in the pyramidal cell layer of dorsal CA1 of the hippocampus, with a diagram of the ABA drug procedure below. ABA trials were delivered 24 h apart over three consecutive days, (b) Mean running speed across the track, excluding the reward region, (c) Mean variability of running speed as the animal runs around the track, (d) Plot of power spectrum density showing reduced theta power in the ketamine condition. Bold colors denote mean power with standard error of the mean in faded color, (e) Mean theta frequency, and (f) Mean firing rate of all putative place cells recorded in CA1. (g) Mean infield firing rates of putative place cells in CA1.

Recent evidence shows that the starting phase of precession is more variable in a developmental model of a schizophrenia risk factor (Speers et al., 2021), potentially disrupting sequential memory and planning mechanisms. If this occurs in schizophrenia, it could account for some of the memory and organizational deficits observed in the disorder. A key question, is therefore, whether acute ketamine also disturbs phase precession in a way that might disrupt sequential processing mechanisms, thereby providing a potential mechanism for some of its schizo-mimetic properties. Previous studies have shown that ketamine alters glutamatergic signaling, PV interneuron density and hippocampal theta activity (Adell et al., 2012; Caixeta et al., 2013; Kittelberger et al., 2012; Koh et al., 2016), and since these mechanisms contribute to the coordination of single cell firing relative to the background theta oscillation (Royer et al., 2012; Wulff et al., 2009), we hypothesize that ketamine should also disrupt phase precession. As far as we are aware, however, no previous study has examined the effect of ketamine on phase precession. In the present study we used an ABA protocol to examine the effects of saline (A) or acute ketamine (7.5 mg/kg; B) administration on hippocampal phase precession in adult male rats (see Figure 1a). It has previously been shown that animals can successfully discriminate this dose of ketamine from saline injections in an operant chamber (Meighan et al., 2021). Single cell and LFP activity from CA1 were recorded as pre-trained animals navigated clockwise around a rectangular running track for a food reward.

## Materials and methods

### Animals

Adult (3–9 month) male Sprague Dawley rats were housed in littermate groups of 1–2 in individually ventilated cages. The housing room was maintained at a normal 12-h light/dark cycle, and temperature controlled to 20–22°C. Animals were food deprived to no less than 85% of their free-feeding weight in preparation for the experimental procedure. All training and testing was conducted during the light phase.

### Apparatus and training

Prior to surgical implantation, animals were trained to run clockwise around a rectangular wooden circuit measuring 900 mm by 800 mm (Figure 1a). All arms were 100 mm wide with 270 mm high side walls. This apparatus is identical to the track described in Speers et al. (2021). A video camera was mounted to the ceiling of the recording room to track the animal’s position. All experiments were performed in a darkened environment with some ambient light from the recording computer and a small lamp aimed away from the apparatus into one corner of the room.

Animals were trained over a period of 5–15 days. On days 1–5, rats were habituated to the recording room and apparatus, and allowed to free-forage for Coco Pops (Kellogg Company) which were scattered throughout the apparatus. Following successful habituation, the placement of Coco Pops was gradually restricted, first to the top two corners of the maze and in the center of the reward arm, and then to the reward arm only. During this period, rats were trained to run in a clockwise direction and were turned back to the correct direction with a paddle when necessary. Coco-pops (approx. 6 per reward delivery) were delivered manually by the experimenter. Training was considered completed when rats consistently ran in a clockwise direction for the food reward over a 20-min session.

### Surgical procedures

**Figure 2.**
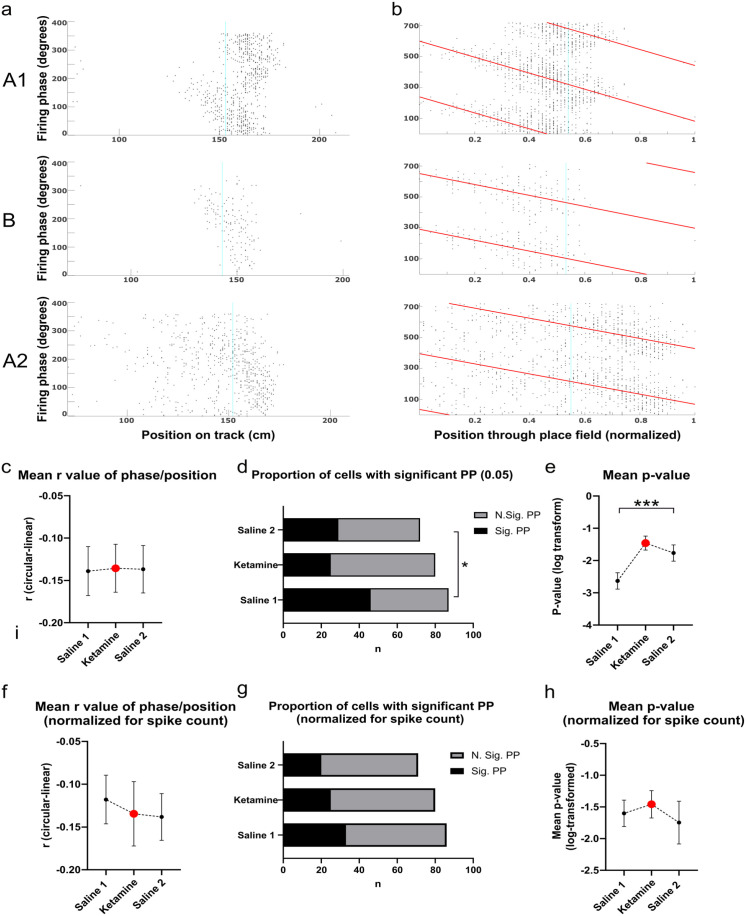
Example phase precession plots and quantification of basic phase precession properties of putative place cells recorded in CA1: (a) Example plots of spiking phase of a single putative cell tracked across an ABA trial, from top to bottom. The x-axis shows the position of the animal across the first 200 cm of the linearized running track, with zero corresponding to the bottom left corner in Figure 1a, and the y-axis shows spike phase, (b) As for a, but with the animal’s position normalized to place field size. Theta phase is duplicated across two theta cycles for visual clarity. The red line denotes the line of best-fit for the circular-linear regression of spike phase and normalized position through the place field, (c) Mean *r* value of the correlation of phase and position across the three experimental groups, (d) Proportion of all putative place cells recorded in CA1 that show significant phase precession, as determined by the *p* value (0.05) of the circular linear correlation of phase and position, (e) Mean log-transformed *p* value of the correlations shown in d, and (f–h) As for c–e, but after the random removal of spikes to normalize for spike number across groups.

All surgical procedures were identical to those described previously in Speers et al. (2021). Animals were anesthetized with 5% isoflurane (Merial New Zealand) in oxygen and maintained at 1.5–2.5% throughout surgery. After animals were deeply anesthetized, they were given a subcutaneous injection of Atropine (1 mg/kg) to ease their breathing, as well as the analgesics Carprofen (1 mg/kg) and Temgesic (buprenorphine; 0.1 mL), and a prophylactic antibiotic, Amphoprim (trimethoprim and sulfamethazine, 0.2 mL). Rats were then mounted on a stereotaxic apparatus (David Kopf Instruments) above a heating pad, and a lubricating eye gel (Visine) was applied. The scalp was shaved and sterilized with Betadine (Povidone-iodine), followed by a subcutaneous injection in the scalp of the local anesthetic Lopaine (lignocaine hydrochloride 20 mg mL^−1^; 0.1 mL diluted in 0.4 mL of saline). After exposing the skull, an opening was drilled above the left hemisphere dorsal hippocampus, and a custom built, eight channel microdrive containing two moveable tetrode bundles of equal length was targeted to the CA1 subregion at −3.8 mm AP from bregma, −2.5 mm ML from the midline, and lowered just above the pyramidal cell layer (1.8 mm from dura; Figure 2b). Electrodes consisted of 25 µm nichrome, heavy formvar insulated wire (Stablohm 675 HFV NATRL; California Fine Wire Company, Grover Beach, CA), and had been gold electroplated until impedances were reduced to ~200–300 kΩ (NanoZ, Neuralynx, Bozeman, MT). Microdrives were secured to the skull with jewelers’ screws and dental cement, and a ground wire was secured to an additional screw placed above the right hemisphere. Post-surgery rats received a secondary dose of Amphoprim immediately upon waking, and then an additional dose of Carprofen 24 h later. Rats were provided with ad libitum food and water post-surgery and were given 8 days to recover. All experimental protocols were approved by the Otago University Animal Ethics Committee and conducted in accordance with New Zealand animal welfare legislation.

### Electrophysiological recordings

Following recovery, rats were again food deprived to no less than 85% of their free-feeding weight. Post-operative training was carried out to ensure that rats could still perform the task adequately, and rarely exceeded a single session. Rats were then attached to a multichannel data acquisition system (DacqUSB; Axona Ltd, St Albans, UK), and cell activity was closely monitored until single units were identified. Electrophysiological recordings of both single cell and LFP data were acquired via a multichannel data acquisition system (DacqUSB; Axona Ltd, St Albans, UK). Extracellular unit activity was first passed through an AC-coupled unity gain amplifier before passing through to the recording system. Single unit data was bandpass filtered between 600 and 6000 Hz, and sampled at a rate of 48 kHz with 24-bit resolution. For each tetrode, one electrode with minimal spiking activity was selected as a reference. Action potential thresholds were set at a minimum of 70–80 µV and recorded for a 1 ms window whenever the spiking amplitude met this threshold. All spike events were time-stamped relative to the beginning of the recording. LFP data was simultaneously recorded from electrodes that had active place cells and were referenced to ground. LFP data was low-pass filtered at 500 Hz (with notch filtering selective for activity at 50 Hz) and sampled at 4800 Hz. The animal’s location was determined from three infrared LEDs mounted on the animal’s headstage and recorded by a camera located above the chamber. Positional data was analyzed with a sampling rate of 50 Hz and then converted into *x* and *y* coordinates by the recording system. Tetrodes were slowly lowered (~40 µm per day) toward the dorsal CA1 pyramidal cell layer until well-isolated single units were identified, as described previously in Speers et al. (2021). Once well-isolated single units were identified, tetrodes were only nudged slightly over the course of the experiment, and only after the completion of a unique trial block. Rats ran no more than one session per day, for ~60–80 laps per session. Single unit, position and LFP data were saved for later analysis.

### Drug treatment

Ketamine hydrochloride 100 mg/ml (Pheonix Pharm, Auckland, NZ) was diluted to a concentration of either 25 mg/ml or 7.5 mg/ml in 0.9% physiological saline, and administered to animals at a dose of 7.5 mg/kg via intraperitoneal injection. The dose was chosen based on data from our previous study Meighan et al. (2021), which indicated that animals could discriminate a dose within the range of 3 and 10 mg/kg from saline, but that this was not the case for lower (1 mg/kg) or higher (30 mg/kg) doses. Furthermore, data from the same study indicated that doses of 3 and 10 mg/kg had no effect on control animal locomotion, whereas the effect of 30 mg/kg was marked. Once single units had been identified, an ABA format was applied to experimental trials. An equivalent amount of saline was administered on day 1 (A1), followed by ketamine administration on day 2 (B), and then another trial of saline on day 3 (A2). The reported half-life of ketamine in rats is approximately 2 h (Veilleux-Lemieux et al., 2013), and so the drug should be largely eliminated with a 24 h delay between the Ketamine and Saline 2 trials. Once subjects had been injected, they were placed back in either their home cage or a transportation box for 15–20 min, as evidence from prior studies indicates that rats may experience initial locomotor difficulties during this period following subanesthetic doses of ketamine (Becker et al., 2003). Our own observations were consistent with this finding, with most rats experiencing locomotor difficulties for approximately 15–20 min post-injection. After the delay, animals were then transported to the recording room and attached to the recording system for experimental trials which lasted from 20 to 30 min. All recordings with at least one putative place cell were included in the final dataset on the condition that a full ABA trial (recorded over 3 days) was completed for that animal. Where animals received repeated ABA sequences, at least 1 week elapsed between each ketamine administration day.

### Data analysis

#### Spike sorting

For each recording, single units were identified manually offline using purpose-designed cluster cutting software (Plexon Offline Sorter, Version 3, Dallas, TX), primarily via the peak-to-valley distance and principal components analysis of the waveforms. Putative place cells were isolated if they had an average firing rate <5 Hz, a peak to trough spike width of ~400 µs, and a complex pattern of bursting activity identified from the autocorrelation of spike times. Cells that did not meet these criteria, such as cells with a distinctive interneuron profile, were excluded from further analysis. Sorted data was then exported to MATLAB (version R2019a, MathWorks, Natick, MA), and analysis of single unit, position and LFP data was carried out in MATLAB with custom-written scripts.

#### LFP analysis

Sampling of LFPs occurred from either the same electrode from which unit data was detected or another tetrode in the same bundle. LFP data were resampled at 1000 Hz and power spectral density (psd) data for each recording were obtained using the MATLAB pwelch routine using a 4096 sample window and 1024 sample overlap. Theta power was obtained from the psd between 6 and 10 Hz either as power at peak, or as area under the curve within this range.

Theta was defined as oscillatory activity that occurred in the 6–10 Hz range. Correlations between theta power and speed, and frequency and speed, were generated for each recording by sampling data at 500 ms intervals. Instantaneous values for theta frequency were estimated from the unwrapped slope of the phase of the Hilbert transform of LFP filtered between 6 and 10 Hz. Estimates of instantaneous speed were determined by monitoring the animals’ change in position over 500 ms time windows, and the mean running speed was then calculated by averaging across bins where the animal was not in the reward region.

Low gamma was defined as oscillatory activity in the 30–60 Hz range, and high gamma was between 60 and 90 Hz. HFOs were those occurring between the frequency range of 130–180 Hz (Olszewski et al., 2013). Evidence of theta/gamma cross-coupling was calculated via the modulation index (MI) according to methods detailed by Tort et al. (2010). First, the raw EEG signal was filtered at the phase and amplitude frequency ranges for theta (6–10 Hz) and either low or high gamma. The phase and amplitude time series were then calculated from these filtered signals using the Hilbert transform. A composite phase/amplitude time series was then constructed, and from this, the mean amplitude distribution over phase bins was obtained. From this distribution, the Kullback-Leibler (KL) distance was computed (Kullback and Leibler, 1951), which is a measure of how much the distribution of EEG amplitude across phase bins deviates from a normal distribution. The MI can be calculated from the following equation:

$$MI=\;\frac{D_{KL}\;\left(P,\;U\right)}{\log\left(N\right)}$$

where *D_KL_* = the KL distance, *P* is the observed amplitude distribution, *U* is the uniform distribution, and *N* is the number of phase bins. Higher MI values indicate more robust cross-coupling.

#### Single cell analysis and phase precession

The rectangular track was linearized so that the starting location was the lower left corner of the running track after the food delivery area (Figure 1a). Place fields were identified by dividing the track floor into 1 cm-long bins and creating an occupancy map from the position tracking data based on the amount of time the rat spent in each bin. Spikes were binned similarly for each single unit by identifying the number of spikes that occurred within each bin. Element-wise division was then used between the spike map and the occupancy map to create a firing mate map where each bin contained the firing rate for a cell. Firing rate maps were smoothed with a 10 cm-wide moving window. Place fields were then detected automatically by detecting regions of at least 10 cm in length that had a firing rate of at least twice the mean firing rate for the cell (Porter et al., 2018). If more than two place fields were detected for a cell, only the largest was analyzed. Following this, each place field map was analyzed separately to determine place field length and mean infield firing rate. Where place fields wrapped around the start-end position of the linearized maze they were linearly shifted prior to firing rate analysis.

Spatial information content provides a measure of how informative a spike from a cell is regarding the animal’s current location within an environment. Place cells with a higher information value therefore provide a more reliable prediction of current location than cells with a lower information value (Skaggs et al., 1993). The formula for information content, measured in bits per spike is:

$$Information=\;\overset{N}{\underset{i=1}{\sum }}p_{i}\;\frac{\lambda_{i}}{\lambda }log2\;\frac{\lambda_{i}}{\lambda }$$

where the environment is divided into *N* distinct bins (*i* = 1, . . ., *N), p_I_* denotes the occupancy probability of bin *i, λ_i_* is the mean firing rate for bin *i*, and *λ* is the overall mean firing rate of the cell.

For all phase precession analyses, the phase reference was always to the LFP signal taken from the CA1 pyramidal cell layer, and 0° corresponds to the trough of the oscillation. For all spikes that occurred within a place field, spike phase was determined by matching the animal’s position within the place field to the instantaneous phase of the 6–10 Hz theta rhythm, and then analyzed using procedures described previously (Kempter et al., 2012). This involves using circular-linear regression to provide a robust estimate of the slope and phase offset of the regression line, and a correlation coefficient for circular-linear data analogous to the Pearson product-moment correlation coefficient for linear-linear data. The fits were constrained to have a slope of no more than −2 and +1 theta cycles per place field transverse. Phase precession analysis was conducted by pooling spiking data from all passes through the place field within a given recording session. Because theta states are associated with locomotion (Vanderwolf, 1969), phase precession analysis was only performed on data where the animal was running at least 5 cm/s. Where normalization was used to match firing rates across conditions, spikes were randomly removed from the data until the overall firing rate of the manipulated file reached the required level.

### Statistical analysis

For all statistical analyses, we performed the following procedure. First, raw data were transformed to a lognormal distribution if appropriate. All data (either in raw form or the log transform) were then checked for assumptions of normality and equality of variances. These checks were performed in GraphPad Prism 8.1.1 (GraphPad Software, Inc., San Diego, CA, USA), using the d’Agostino and Pearson test for normality, and the F test to compare variances. If data did not meet the assumptions for normality based on the d’Agostino and Pearson test, visual inspection of histograms and QQ plots was performed, and extreme outliers were removed using the Graphpad function for removal of outliers. All data that failed to meet assumptions of normality based on this procedure were then analyzed using the appropriate non-parametric test. All *t*-tests were two-tailed. Data with a normal distribution are presented as mean ± SEM unless explicitly stated otherwise in the figure legends. Significance levels were defined as *p* < 0.05. Additional information about significance levels is provided in the figures as: *p < 0.05, **p < 0.01, ***p < 0.001.

### Histology

Following completion of experiments, rats were anesthetized with 5% isoflurane in oxygen, and a 2 mA direct current was passed through each electrode for approximately 1 s to lesion the site of the electrode tip. Rats were then euthanized with an overdose of isoflurane and transcardially perfused, first with 120 ml of 0.9% saline, and then 120 ml of 10% formalin in saline. Brains were then carefully extracted from the skull after removal of the Microdrive, and stored in 10 % formalin in saline. One week prior to sectioning, brains were transferred first to 10% formalin in H_2_O for 24 h, and then to a 10% formalin/30% sucrose solution for approximately 3–7 days, until the brain sunk to the bottom of the sucrose solution. Dehydrated brains were then sectioned into 60 μm coronal slices with a cryostat (Leica CM1950). Sections were then mounted on slides and stained with a thionine acetate Nissl stain (Santa Cruz Biotechnology, Inc., Dallas, TX) After slides were dry (min. 24 h) electrode placement was imaged with a local power (1.5×) digital microscope (Leica Biosystems, LLC, Deer Park, IL) to verify electrode placement.

## Results

### Acute ketamine administration decreases running speed, single unit firing rates and both theta power and frequency relative to saline controls

Each ABA manipulation was conducted over three consecutive days, with one ABA block per week, per animal, for a maximum of 6 weeks, or until cells were no longer recorded. This resulted in 21 ABA blocks recorded across seven animals. In terms of behavior, mean running speed was significantly reduced following ketamine administration relative to the pre- and post-saline conditions (*F*(2,20) = 4.93, *p* = 0.016, repeated measures; Figure 1b). The variability of locomotor speed within a single recording was also reduced under ketamine, as calculated by the mean standard deviation of speed sampled every 500 ms (*F*(2, 20) = 15.59, *p* < 0.0001, repeated measures; Figure 1c).

Inspection of power spectral density plots showed that power was reduced specifically in the theta frequency range under ketamine (Figure 1d), and peak power within the theta band was significantly different when comparing the mean of peak power pre- and post-ketamine to ketamine trials (*t*(20) = 2.26, *p* = 0.036, repeated measures). Ketamine also reduced the frequency of the LFP theta (6–10 Hz) oscillation (*t*(20) = 3.71, *p* = 0.0014). To determine whether ketamine changes the relationship of either theta power or frequency to speed, we next computed the correlation *r* values between power and speed, and frequency and speed within each recording, from data sampled every 500 ms. The mean *r*-value of this correlation was significantly different from zero for all conditions (Saline 1 mean *r* = 0.07, *t*(20) = 3.09, *p* = 0.0057, Ketamine mean *r* = 0.08, *t*(20) = 3.94, *p* = 0.0008, Saline 2 mean *r* = 0.06, *t*(20) = 3.63, *p* = 0.0017), indicating there was a weak relationship between these variables across all conditions. The mean *r* value for power/speed was not, however, significantly different between groups (*F*(1.64,32.88) = 1.05, *p* = 0.349, repeated measures, Greenhouse-Geisser corrected). Similarly, the mean *r* value of the correlation of theta frequency (as determined from the Hilbert transform) and speed was significantly different from zero across all conditions (Saline 1 mean *r* = 0.30, *t*(20) = 11.07, *p* < 0.001, Ketamine mean *r* = 0.24, *t*(20) = 7.03, *p* < 0.001, Saline 2 mean *r* = 0.27, *t*(20) = 8.46, *p* < 0.001). There was a significant reduction in the mean *r* value of the correlation of theta frequency and speed under ketamine (*F*(1.85, 37.06) = 3.71, *p* = 0.037, repeated measures, Greenhouse-Geisser corrected), suggesting that ketamine may weaken this relationship to some extent, although it does not abolish it. To estimate the extent that the change in locomotion speed might have affected theta power and frequency we used the slope of the regression line of the speed-frequency and speed-power linear best-fit relationship to predict what change would be expected in theta as a result of the reduction in locomotion speed. This analysis showed that the reduction in theta frequency was as predicted by the reduction in speed. However, the reduction in power was an order of magnitude greater than was predicted by the reduction in speed, indicating that ketamine was reducing theta power to a greater extent than was expected from the change in locomotion alone.

We did not observe any ketamine effects in LFP power or peak frequency outside the theta band. In particular, no change was observed in either the low (40–60 Hz), high gamma (60–90 Hz) ranges (low gamma *t*(20) = 0.02, *p* = 0.981; high gamma *t*(20) = 0.14, *p* = 0.891), or HFO (*t*(20) = 0.624, *p* = 0.539) frequency bands. There were also no significant differences for hippocampal theta/gamma cross-frequency coupling in either the low or high gamma bands (high band, repeated measures *F*(2,20) = 0.14, *p* = 0.837; low band, repeated measures *F*(2,20) = 0.30, *p* = 0.719). Except where mentioned otherwise, the ketamine effects observed on the first day of administration were similar to those observed on subsequent ABA sequences.

In total, 239 putative place cells were recorded from the hippocampus, spread across seven animals and 63 recordings (21 unique ABA blocks). Of these, 87 units were recorded during the first saline (A1) condition, 80 in the ketamine condition (B) and 72 in the second saline condition (A2). Both the overall mean and the infield firing rates were significantly reduced under ketamine (mean firing rate, *F*(2,236) = 4.91, *p* = 0.008; infield firing rate, *F*(2,236) = 4.32, *p* = 0.014, Figure 1f, g). Infield firing rates were significantly correlated with running speed (as measured at the time of the spike) for all groups (A1 *r* = 0.42, *p* < 0.001); B *r* = 0.36, *p* = 0.001; A2 *r* = 0.48, *p* < 0.001), although mean firing rates were only significantly correlated with speed (as measured across the entire track) in the saline conditions (A1 *r* = 0.35, *p* = 0.001; B *r* = 0.20, *p* = 0.08; A2 *r* = 0.37, *p* = 0.001). Because speed is known to affect the firing rate of place cells, we performed an analysis of covariance (ANCOVA) for infield firing rates with speed as a covariate. This analysis showed that locomotor speed accounts for the difference in firing rates, as the group effect was no longer significant (*F*(2,235) = 2.24, *p* = 0.109, partial *η*^2^ = .02).

### Acute ketamine administration does not alter place field size but alters phase precession slope

Ketamine did not alter either the mean place field length relative to saline trials, *F*(2, 236) = 0.59, *p* = 0.557) or the mean information content as measured by bits per spike (*F*(2,259) = 0.22, *p* = 0.805). Phase precession was measured by computing the circular-linear correlation of spike phase and normalized position as an animal traversed a place field (see Figure 2a and b for example plots). Spikes recorded when the animal was running less than 5 cm/s (such as when the animal was consuming the reward or grooming) were omitted from the analysis. Initial analyses indicated that ketamine disrupted the proportion of place cells showing significant phase precession (as defined by the *p*-value 0.05) of the correlation of phase and position, (χ^2^(2) = 8.11, *p* = 0.017). In total, 53% of cells significantly precessed in the saline 1 condition, while only 31% and 40% of cells significantly precessed in the ketamine and saline 2 conditions respectively (Figure 2d). Analysis of the log-transformed *p*-values also showed a significant group effect (Kruskal-Wallis statistic = 15.43, *p* = 0.0004, Figure 2e). There was no difference for the mean *r*-value of the circular correlation when all cells were included in the analysis (S1 *M* = −0.139 ± 0.029, K *M* = −0.136 ± 0.028, S2 *M* = −0.137 ± 0.028, *F*(2, 236) = 0.003, *p* = 0.996, Figure 2c), or when only the subset of cells showing significant phase precession were included (S1 *M* = −0.201 ± 0.050, K *M* = −0.357 ± 0.052, S2 *M* = −0.265 ± 0.053, *F*(2,97) = 2.12, *p* = 0.126).

Given that ketamine could potentially induce chronic effects across repeated trial blocks, these data were also analyzed with the subset of cells recorded during the first ABA trial block. These results were generally consistent with analysis of the entire data set, with a lower proportion of cells showing significant phase precession following the ketamine injection (Saline 1 = 58%, *n* = 31, Ketamine = 35%, *n* = 31, Saline 2 = 45%, *n* = 22). However, this difference was no longer significant, *χ*^2^(2) = 3.19, *p* = 0.203, and mean *p*-values (log-transformed) in this reduced dataset were only trending toward significance (Kruskal Wallis statistic = 5.97, *p* = 0.051). The mean *r*-value did not differ between groups (*F*(2, 81) = 0.26, *p* = 0.770).

Another possibility is that the reduction in significantly phase precessing cells that we observed under ketamine is an artifact of reduced firing rates. To test this possibility, firing rates in both saline groups were artificially reduced by randomly removing spikes so that the mean spike count for each group was equivalent. This procedure resulted in a lower proportion of significantly precessing cells in both the Saline 1 and Saline 2 groups, so that now around one-third of cells in every group showed evidence of significant precession (Figure 2g). These proportions were not significantly different between groups (*χ*^2^(2) = 1.96, *p* = 0.371), which suggests that the lower number of spikes under ketamine likely accounts for the lower incidence of significant phase precession in this group. The mean, log-transformed *p*-value of the circular-linear correlation of phase and position was also no longer significant (Kruskal Wallis statistic = 1.89, *p* = 0.3884, Figure 2h).

**Figure 3.**
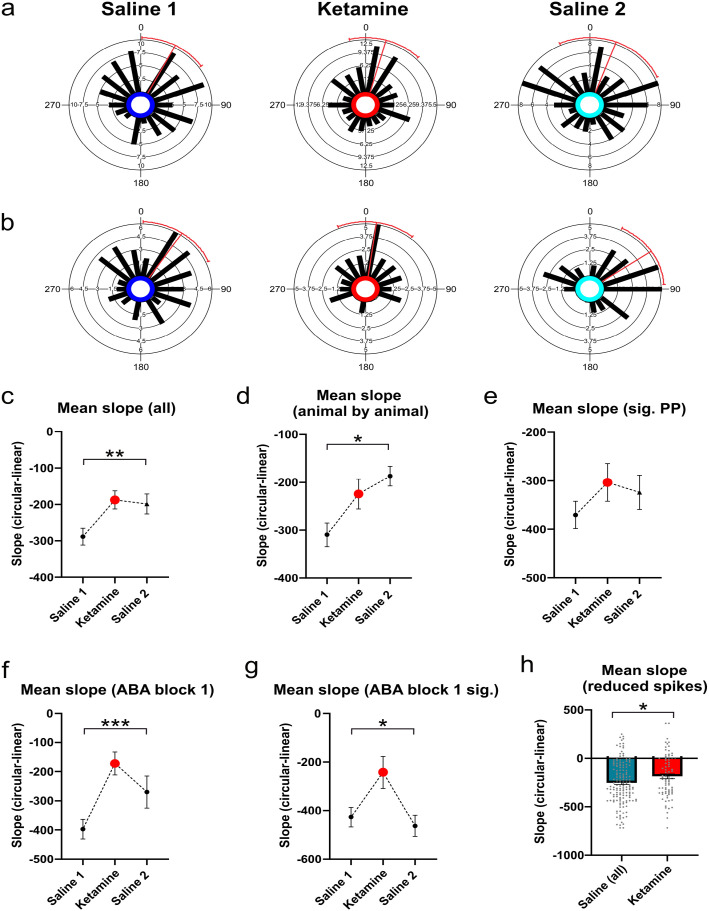
Phase precession starting phase and range, as measured by the intercept of slope of the circular linear correlation of phase and position respectively: (a) Circular histograms of starting phase for all putative place cells recorded in CA1, (b) As for a, but with only the subset of cells showing significant (0.05) phase precession, (c) Mean regression slope of the circular-linear correlation of phase and position for all putative place cells, (d) Mean slope on an animal by animal basis, (e) Mean slope for the subset of cells showing significant phase precession, (f) Mean slope for all cells recorded during the first ABA block for each animal only, (g) As for f, but only for the subset of cells showing significant phase precession, and (h) Mean slope after the random removal of spikes to normalize for firing rates across groups. Pre- and post-ketamine A trials have been collated into a single saline dataset.

Ketamine did not significantly affect the starting phase of precession as an animal enters a new place field, as measured by the intercept of the circular-linear correlation of phase and position. In the saline 1 condition, the intercept phase was significantly clustered around a mean angle of 27.67° (Rayleigh *Z* = 8.26, *p* = <0.001), in the ketamine condition it was significantly clustered around a mean angle of 16.09° (Rayleigh *Z* = 7.20, *p* = <0.001), and in the Saline 2 condition it was significantly clustered around a mean angle of 20.61° (Rayleigh *Z* = 3.10, *p* = 0.045, Figure 3a). A Mardia-Watson-Wheeler test to compare across groups indicated that these different phase angles were not significantly different from each other (*W* = 2.51, *p* = 0.643). Similar results were obtained when only the subset of cells showing significant phase precession were included, with intercept values in the saline 1 condition significantly clustered around a mean angle of 33.74° (Rayleigh *Z* = 5.77, *p* = 0.003), 8.43° in the ketamine condition (Rayleigh *Z* = 5.94, *p* = 0.002) and 55.77° in the saline 2 condition (Rayleigh *Z* = 6.14, *p* = 0.002, Figure 3b). The Mardia-Watson-Wheeler test was also not significant (*W* = 6.37, *p* = 0.173).

The slope of phase precession describes how much phase of firing shifts as an animal moves through a cell’s place field, and thereby provides an estimate of the range of precession across a single theta cycle. When these data were examined, there was a significant group difference for the slope of the circular-linear correlation, with cells in the ketamine group showing shallower slopes when compared to cells in the saline 1 condition (*F*(2, 236) + 5.18, *p* = 0.006, Figure 3c). Mean slopes were also calculated on an animal by animal basis, and a repeated measures analysis of variance (ANOVA) showed that mean slope was significantly shallower under ketamine (*F*(2,6) = 5.95, *p* = 0.019, Figure 3d). These effects were no longer significant when the smaller group of cells that showed significant phase precession were analyzed (Kruskal Wallis statistic = 3.34, *p* = 0.188, Figure 3e). Precession slope was, however, significantly reduced following ketamine administration when analysis was limited to cells recorded in animals receiving their first ketamine dose, both for all cells (*F*(2, 81) = 8.25, *p* = 0.0005, Figure 3f) and for the subset of cells showing significant phase precession (*F*(2,36 = 4.92, *p* = 0.013, Figure 3g). The group difference for slope was still evident following random spike reductions made to normalize firing rate across the three experimental conditions (although the ANOVA was borderline (*F*(2,234) = 2.96, *p* = 0.054)). When the two saline conditions (A1 and A2) were combined, however, and a t-test was used to compare all saline trials to ketamine trials, mean slope under ketamine was significantly reduced (*t*(232) = 2.22, *p* = 0.027, Figure 3h). In the saline 1 condition, when data from all cells are included and averaged, cells precess across a large range of the theta cycle (289°) as an animal runs through a place field. In contrast, in the ketamine condition, precession spanned only around one half of the theta cycle (187°).

## Discussion

Our data show that the acute administration of a subanesthetic dose of ketamine reduces the power of theta-frequency LFP activity and decreases the range of precession across a theta cycle as an animal runs across a place field. While ketamine also reduced the infield firing rate of place cells, and the frequency of theta, these changes appeared to be associated with the reduction in locomotor speed that was also observed, consistent with previous reports (Czurkó et al., 1999; Fuhrmann et al., 2015; Geisler et al., 2007; McNaughton et al., 1983). The directionality of the theta frequency to locomotor speed relationship is unclear; however, it is possible that ketamine affects speed by increasing the regularity of hippocampal theta oscillations (Bender et al., 2015).

Although ketamine reduced the range of phase precession, other aspects of the phenomenon were preserved. This included features such as the starting phase of precession and the clustering of spiking around the mean slope of precession. This indicates that the base phenomenon of phase precession persevered following acute NMDA receptor antagonism and is consistent with previous data showing that phase precession is relatively unaffected by fluctuations in locomotion speed (Geisler et al., 2007). Our findings also demonstrate that phase precession can be maintained under conditions where theta power and cell firing rates are reduced. Although the reductions in theta power, theta frequency and firing rates that occurred were similar to those described in previous studies (Caixeta et al., 2013; Kittelberger et al., 2012; Kuang et al., 2010; Lazarewicz et al., 2010; Masuda et al., 2023), we did not observe any differences in gamma power, theta/gamma cross-coupling, or HFO power. This was surprising given that several previous studies have observed gamma range disruptions in humans (Curic et al., 2021; Hong et al., 2010) and animal models (Kittelberger et al., 2012; Lazarewicz et al., 2010), as well as disturbed theta/gamma cross-coupling under ketamine (Ahnaou et al., 2017; Caixeta et al., 2013; Michaels et al., 2018). Furthermore, NMDA receptor antagonists have also resulted in increased HFO power in both animal (Olszewski et al., 2013) and human (Nottage et al., 2023) studies. These effects are likely due to methodological differences, as most ketamine studies using animal models have recorded cellular activity while animals explored an open arena, while our animals were pre-trained to run in a stereotyped fashion. One study that required animals to navigate around a familiar track for a food reward similar to our design did observe increased amplitude in the low gamma range, but they used a chronic rather than acute ketamine protocol (Michaels et al., 2018). Consistent with the possibility that ketamine effects are state-dependent, recent evidence shows that the modulating effects of drugs such as clozapine on ketamine-induced activity are far greater when freely moving rats are in a passive state (Bowman et al., 2022).

Ketamine reduced the range of phase precession by approximately 35%. Although it is unclear what effects this reduced range of precession would have on cognition and/or behavior, there are several possible outcomes. First, it would reduce the time between spiking of individual cells that had adjacent place fields and that were firing in a theta sequence. This would increase the risk of disordered spiking, in the sense that the likelihood of two or more cells firing out of the order of their place field sequence would increase. As a result, theta sequences would be less organized, potentially encoding less information about the temporal or spatial order of experience. In some respects, the consequences of this effect are similar to those we observed in the MIA model of a schizophrenia risk factor (Speers et al., 2021). In this previous study MIA increased the variability of the starting phase of precession; however, in both cases the consequence would be a decrease in the accuracy of sequential ordering during the theta sequences that may underlie aspects of sequential memory. While impairments in temporal processing and sequential ordering are well documented in individuals with schizophrenia, their first-degree relatives, and other at-risk individuals (Ciullo et al., 2016, 2018; Liu et al., 2020; Nour et al., 2021; Thoenes and Oberfeld, 2017), the effects of ketamine on such processes are less well investigated (Brulé et al., 2021; Coull et al., 2011; Sawagashira and Tanaka, 2021). Ketamine is, however, known to induce several symptoms of schizophrenia in humans that could potentially involve a sequential component (Malhotra et al., 1996; Morgan et al., 2004).

A reduced range of phase precession may also have an effect on synaptic plasticity mechanisms. With a reduction in precession slope, two cells with adjacent place fields would fire within a narrower temporal window during a theta sequence, which may strengthen potentiation between them (Bi and Poo, 1998). One consequence of this might be an expansion of place field size as cells gain greater influence over the firing of their neighbors (Shen et al., 1997; Terrazas et al., 2005); however, we saw no evidence of this occurring. While ketamine administration has previously been associated with both spatial memory deficits (McKendrick et al., 2020; Morgan et al., 2014; Rowland et al., 2005), long-term depression in the hippocampus (Duan et al., 2013) and diminished LTP (for a review, see Morris and Davis, 1994), improved learning has also been observed in some instances (Shi et al., 2020), particularly among participants diagnosed with depression (Gill et al., 2021; Lara et al., 2013). The latter effect could possibly relate to another consequence of a reduction in precession slope. For most of a theta cycle, phase precession links place cells together in a temporal sequence that recapitulates the animal’s progress through a series of place fields, starting from behind the animal’s direction of locomotion and moving to a position in front of it in each theta cycle. When the range of phase precession approaches 360°, however, it becomes unclear where one sequence/cycle ends and the next one begins. It also means that at the edge of every theta cycle there is a period where cells with place fields that the animal is just entering will fire ahead of those that the animal is leaving. As a result, there is the potential for connectivity to occur that would link cells in the reverse order to the experienced sequence. While such a mechanism might be useful in processes such as reward attribution, it could also hamper normal sequential learning. When the range of phase precession is decreased, as occurs with ketamine, this “reverse learning” is less likely to occur, as there will be a greater phase difference in the firing associated with one theta cycle and the next. This could possibly enhance forward sequence learning, and although this idea is still speculative at this stage, it is an interesting possibility given recent findings showing that, under some circumstances, ketamine can be used for therapeutic benefit (Gill et al., 2021; Mkrtchian et al., 2021; Parise et al., 2013; Riggs and Gould, 2021).

Finally, if the firing phase of hippocampal place cells is used by the brain to encode distance traveled, then a change in phase precession range may have some effect on how the brain perceives distance. For example, when the animal moves across a certain distance, the effect of ketamine might be that the phase precession system would report that only 65% of the actual distance was covered. If other brain systems are monitoring effort expenditure in order to compute cost-benefit, then the effort distance ratio will be changed to indicate that this movement is more effortful than occurs without ketamine (same effort but less distance). This may then affect motivation systems. Consistent with this idea, a previous study has found that phase precession slopes decrease when an animal runs up a tilted track that requires increased effort to traverse (Porter et al., 2018). Ketamine has also been shown to impact motivation in human participants, with improved motivation observed in participants diagnosed with depression (Lally et al., 2015; Mkrtchian et al., 2021), while impaired motivation is observed in healthy control participants (Driesen et al., 2013; Nugent et al., 2019; Pollak et al., 2015).

There are relatively few previous studies that have investigated the effects of pharmacological manipulations on phase precession. These studies have shown, however, that phase precession is disrupted following administration of the cholinergic antagonist scopolamine (Newman et al., 2017; Venditto et al., 2019) and by a cannabinoid receptor agonist (Robbe and Buzsáki, 2009). In the latter case, the slope of the circular-linear correlation was also significantly reduced, similar to the findings reported here (Robbe and Buzsáki, 2009). It is interesting to note that, in humans, both of these compounds produce effects on cognition and experience that overlap with the effects of ketamine.

Previous studies have shown that the temporal coordination of spiking activity may be dependent on PV+ interneuron signaling (Royer et al., 2012; Wulff et al., 2009). Given that both schizophrenia and ketamine exposure have been associated with reduced density of PV+ interneurons (Kittelberger et al., 2012), it is possible that the effects we observed could be a result of changes in these cells. It is also possible that while acute administration of ketamine resulted in only subtle changes to hippocampal phase precession, chronic ketamine use (Ahnaou et al., 2017; Kittelberger et al., 2012), or exposure to ketamine during critical developmental periods, may have more profound effects. For example, selective deletion of NMDA receptors from predominantly PV+ interneurons during early development has been shown to reproduce several of the molecular, physiological and behavioral phenotypes reminiscent of schizophrenia, while similar procedures post adolescence failed to produce the same effects (Belforte et al., 2010). This suggests that the developmental refinement of excitatory/inhibitory circuitry may be more critical for schizophrenia-like outcomes than acute disruption of NMDA receptors. One potentially interesting avenue for future research would be to compare the effects of chronic ketamine delivered during early development on phase precession post-adolescence to determine whether this procedure reproduces the specific disturbances to phase coding at the network level previously observed in MIA animals (Speers et al., 2021). In proposing this, however, we are aware that there were minimal differences observed in our study when we compared the effects in animals that had received their first injection of ketamine with those that had received several over repeated ABA trials.

## Conclusion

Our data indicate that phase precession in the CA1 region of the hippocampus is largely unaffected, despite changes in place cell firing rates, LFP theta properties and locomotor speeds, following an acute, subanesthetic dose of ketamine. Although phase precession persisted under ketamine, however, the range of precession across a single theta cycle was reduced. This reduction in range could potentially modify synaptic plasticity, the representation of the temporal structure of event sequences, spatial learning and memory, and reward motivation. These effects may be a factor in findings that ketamine has cognitive and motivational benefits for people with depression, as well as its ability to mimic several symptoms of schizophrenia in healthy individuals.
